# Decision-making strategies for reperfusion therapies: navigating through stroke trials gaps

**DOI:** 10.1590/0004-282X-ANP-2022-S123

**Published:** 2022-08-12

**Authors:** Mateus Paquesse Pellegrino, Felipe Borelli Del Guerra, Iago Navas Perissinotti

**Affiliations:** 1 Universidade de São Paulo, Faculdade de Medicina, Hospital das Clínicas, Instituto de Radiologia, São Paulo, SP, Brazil.; 2 Hospital de Base do Distrito Federal, Brasília, DF, Brazil.; 3 Universidade de São Paulo, Faculdade de Medicina, Hospital das Clínicas, Instituto do Câncer do Estado de São Paulo, São Paulo, SP, Brazil.; 4 Universidade de São Paulo, Faculdade de Medicina, Hospital das Clínicas, Instituto Central, Departamento de Neurologia, São Paulo, SP, Brazil.

**Keywords:** Stroke, Ischemic Stroke, Thrombectomy, Mechanical Thrombolysis, Thrombolytic Therapy, Acidente Vascular Cerebral, AVC Isquêmico, Trombectomia, Trombólise Mecânica, Terapia Trombolítica

## Abstract

Despite there being a robust amount of literature and numerous randomized clinical trials regarding acute ischemic stroke treatment, the trials have not included some frequent controversial situations for which decision-making strategies are an urgent and unmet need in clinical practice. This article tries to summarize the current evidence about some selected situations (mechanical thrombectomy in low ASPECTS, low NIHSS with proximal occlusion, acute basilar occlusion, distal and medium vessel occlusion, among others), make suggestions on how to approach them in clinical practice and show what to expect in acute stroke research in the near future.

## RESUMO

Apesar de possuir um corpo de literatura robusto e inúmeros ensaios clínicos randomizados sobre o tratamento do AVC isquêmico agudo, os *trials* não incluíram algumas situações frequentes e controversas para as quais as estratégias de tomada de decisão são uma necessidade urgente na prática clínica. Este artigo tenta resumir as evidências atuais sobre algumas situações selecionadas (trombectomia mecânica em ASPECTS baixo, NIHSS baixo com oclusão proximal, oclusão basilar aguda, oclusão de vaso distal e médio, entre outras), propor sugestões de como abordá-las na prática clínica e mostrar o que esperar na pesquisa de AVC agudo no futuro próximo.

## INTRODUCTION

Acute ischemic stroke treatment has been one of the neurology subjects with the most advances in recent years and with the greatest volume of evidence from well-designed randomized clinical trials and extensive guidelines. However, a multitude of not uncommon clinical scenarios have not been properly addressed in the trials and evidence-based guidance on how to manage reperfusion therapies in the hyperacute setting is still lacking in these situations. The objective of this article is to perform a review of the current evidence regarding reperfusion therapy in controversial scenarios and make suggestions that could help the decision-making process in some of these challenging situations.

## ENDOVASCULAR THROMBECTOMY IN LOW ASPECTS

Most of the clinical trials that have demonstrated endovascular thrombectomy’s (EVT) benefit in anterior circulation ischemic stroke used ASPECTS (Alberta stroke program early computed tomography score) to select patients and were selective, excluding patients with a score < 6 and presumably a greater core volume. Therefore, little data regarding this population is available from the studies included in the HERMES collaboration^1^.

Although the MR CLEAN trial did not use ASPECTS or ischemic core volume as an exclusion criterion, the median ASPECTS in the study was 9 (IQR 7-10), only 28 (5.6%) out of 496 patients had an ASPECTS < 5 and 120 (24.2%) < 8^2^. 

A pre-specified (but not registered) meta-analysis from the HERMES collaboration that used individual data from seven clinical trials showed a benefit for patients with ASPECTS 3-5, with 30/98 (31%) of EVT group patients achieving a modified Rankin Scale (mRS) of 0-2 at 90 days compared to 14/90 (16%) of the control group (adjusted OR 4.27 [1.62-11.25]), but no benefit for patients with ASPECTS 0-2. Despite the supposed benefit, thrombectomy yielded a greater risk of symptomatic intracranial hemorrhage (sICH), present in 14/95 (15%) of the EVT group and 3/87 (3%) of the control group of patients with ASPECTS 3-5 (OR 4.84 [1.27-27.03]). Similar results of efficacy and sICH were found within the group with initial involvement greater than one third of the middle cerebral artery territory^3^.

After more widespread adoption of stroke thrombectomy, several observational studies were performed trying to assess this matter and they mainly suggest that thrombectomy could confer a better functional outcome in three months compared to conservative management, with some studies also suggesting lower mortality and craniectomy rates. Despite that, some studies also showed augmented risk for symptomatic intracranial hemorrhage^4^ ^-^ ^7^. One retrospective analysis of the German stroke registry also suggested higher risk for sICH, as well as higher mortality in the EVT group and no difference in favorable outcomes. However, a significant effect regarding recanalization status was shown, with 15% of mTICI (modified Thrombolysis in Cerebral Infarction Scale) 0-2a achieving a 90-day mRS 0-3 against 28-36% in mTICI 2b-3^8^.

Two meta-analyses on the effect of EVT in patients with ASPECTS < 6 were performed, the first including 17 studies and 1,378 patients^9^, the second, nine studies and 1,196 patients^10^. Both suggested better functional outcomes at 90 days in the endovascular group compared to best medical management, with 30.1% of the EVT group achieving an mRS 0-2 versus 3.2% of the medical group (P=0.001) in the first study^9^ and 27.7% and 3.7% in the second study, respectively (P=0.001)^10^. Regarding sICH, they produced conflicting results, one suggesting lower odds in EVT group (20% vs 31.7%, P=0.05)^9^, while the other showed a trend towards higher rates (9.2% vs 5.5%, P=0.11)^10^. One of the meta-analyses produced some subgroup data, showing that patients younger than 70 had higher rates of mRS 0-2 compared to older patients (40.3% vs 16.2%) and that initial ASPECTS had a direct relation with 90-day mRS 0-2 (33.3% of ASPECTS 5, 22.1% of ASPECTS 4, 13.9% of ASPECTS 0-3)^9^. Despite these important findings, both meta-analyses had serious limitations: most of the included studies were retrospective, not randomized and several lacked a control group; heterogeneity was high for most studied outcomes; the definition of sICH was variable among studies; ASPECTS could be determined using magnetic resonance imaging (MRI) or computed tomography (CT) depending on the study. 

Despite ASPECTS being used as a selection criterion in most trials and guidelines until now, its use has great limitations, as each of the 10 predefined areas have diverse volumes and eloquence and each point is deducted in a binary fashion, disregarding the infarcted volume within each region, the degree of hypodensity and the eloquence of unaffected areas within each ASPECTS region. Some studies have already showed that the correlation of ASPECTS and core volume is limited^11^ and the more focused analysis of eloquence and degree of involvement in each region might play an important role in functional outcome and treatment effect^12^.

A recent single center observational study showed that in patients with ASPECTS ? 5 and baseline infarct volumes ? 70 ml the outcome of mRS 0-2 at 90 days in patients submitted to EVT was 38.9% against 18.8% of patients with volumes > 70 ml (P=0.04 after adjusted multivariate analysis)^13^. A post-hoc analysis of a HERMES collaboration meta-analysis that included 177 patients with baseline large core (defined as 80-300 ml in diffusion-weighted MRI / CT-perfusion or ASPECTS ? 5) suggested that in patients with cores 80-130 ml or ASPECTS 4-5, thrombectomy was associated with functional improvement (OR 2.11 [95% CI, 1.08-4.09]), but that could not be demonstrated in patients with cores > 130 ml or ASPECTS ? 3 (OR 1.75 [95% CI, 0.62-4.89]), in which EVT was also associated with possible worsening of edema and greater midline shift in follow-up images^14^. Both studies, despite their limitations, reinforce the importance of evaluating volumes and not only ASPECTS in the treatment decision.

Probably the best piece of evidence until now is the recently published randomized clinical trial conducted in Japan (RESCUE-Japan LIMIT), which randomized 203 patients with ASPECTS 3-5 to receive EVT or best medical care within six hours after they were last known to be well or within 24 hours if there were no early changes in FLAIR (fluid-attenuated inversion recovery) images. Patients had to have at least 6 points in the NIHSS, a previous mRS ? 1, occlusion of internal carotid artery or M1 segment of middle cerebral artery. ASPECTS could be measured either with CT or DW-MRI. No relevant baseline disparities were seen between groups and in general the included patients consisted of moderate to severe strokes: median NIHSS of 22, median ASPECTS of 3, median infarct volume of 94 ml (IQR 66-152) in EVT group and 110 ml (IQR 74-140) in the medical care group. The primary outcome of mRS 0-3 at 90 days was achieved in 31.0% of the endovascular group and 12.7% in the medical care group (RR 2.43; 95% CI 1.35 to 4.37; P=0.002). Any intracranial hemorrhage within 48 hours was significantly higher in EVT group (58% vs 31.4% [RR 1.85, 95% CI 1.33-2.58, P<0.001]) and sICH was also numerically higher, although not statistically significant (9.0% vs 4.9% [RR 1.84, 95% CI 0.64-5.29, P=0.25]). The main limitations of the study are that treating physicians and patients could not be blinded, the population consisted only of Japanese patients, alteplase was used in a small percentage of patients (27%), the standard dose of alteplase used in Japan is 0.6 mg/kg and almost 90% of ASPECTS were calculated using MRI, limiting the applicability of the results to ASPECTS calculated using CT^15^.

Besides achieving reperfusion of potentially salvageable and eloquent areas, other mechanisms of possible benefit of EVT in large stroke patients might play an important role, such as diminishing associated cerebral edema^7^ and preserving vascular cells in the ischemic area, facilitating vascular and neural repair^16^.

With the existing evidence, EVT in patients with ASPECTS 0-2 seems to be futile, but it might be a reasonable treatment option for patients with ASPECTS 3-5, especially in the following situations: ASPECTS measured using diffusion-weighted MRI, ASPECTS 4-5, ischemic core volume < 130 ml (even better if < 70 ml), younger age, less edema and spared eloquent areas in the baseline image. However, several controversies still remain: the augmented risk of hemorrhagic transformation; no consensus about the best image modality for optimal selection (CT, CT-perfusion, diffusion-weighted MRI or novel techniques to measure established edema as baseline Net Water Uptake)^17^; the cost-effectiveness of the treatment, especially in a resource limited setting; better definition of a cutoff volume or ASPECTS for which benefit would still be sustained; establish the role of area eloquence analysis, age, collateral circulation status, beyond other factors. 

Several clinical trials of EVT in low ASPECTS/large core are ongoing, with more data expected to be added in the next months/years (Table 1).

**Table 1.** Ongoing randomized clinical trials regarding main themes addressed in the article^80^.

Study	Comparison	Estimated enrollment	Inclusion criteria	Primary outcome	Country/region	Estimated completion
Low ASPECTS / Large Core						
LASTE (NCT03811769)	Thrombectomy vs medical care	450	ASPECTS (CT or DWI) 0-5 if <80 years or 4-5 if ≥ 80 years; < 7h from LKW; ICA (isolated cervical occlusion excluded), M1 or M1-M2 occlusion	180-day mRS shift analysis / 90-day mortality	France	Feb/2022
SELECT-2 (NCT03876457)	Thrombectomy vs medical care	560	ASPECTS (CT) 3-5 and/or core volume ≥ 50 ml on CTP/DWI; < 24h from LKW; ICA or M1 occlusion	90-day mRS shift analysis / 90-day mRS 0-2	USA, Canada, Australia, New Zealand and Spain	Nov/2022
TESLA (NCT03805308)	Thrombectomy vs medical care	300	ASPECTS (CT) 2-5, < 24h from LKW, ICA (cervical excluded) or M1 occlusion	UW 90-day mRS	USA	Nov/2022
ANGEL-ASPECT (NCT04551664)	Thrombectomy vs medical care	488	ASPECTS (CT) 3-5 or core volume 70-100 ml on CTP/DWI in patients with >6h or <6h and ASPECTS (CT) 0-2; > 24h from LKW; terminal ICA or M1 occlusion	90-day mRS	China	Nov/2022
TENSION (NCT03094715)	Thrombectomy vs medical care	665	ASPECTS 3-5 (CT or DWI); < 12h from LKW; M1/ICA occlusion	90-day mRS shift analysis	Europe and Canada	Sep/2024
Distal / medium vessel occlusion						
DISCOUNT (NCT05030142)	Thrombectomy vs medical care	488	NIHSS ≥ 5; < 6h from LKW; primary occlusion of distal M2, M3, P1, P2, P3, A1, A2 or A3	90-day mRS 0-2	France	Dec/2023
DISTALS (NCT05152524)	Thrombectomy vs medical care	168	Perfusion lesion (CTP or MRP) ≥ 10 ml; core (CTP or DWI) ( 50% of perfusion lesion; NIHSS 4-24 or 2-24 if aphasia or hemianopia; <24h from LKW; disabling deficit; primary non-dominant M2, M3, ACA, PCA occlusion and vessel diameter ≥ 1.5 mm; not eligible for IVT	Successful reperfusion (CTP or MRP) and no sICH	Not informed (sponsored by a USA located company)	Aug/2024
DISTAL (NCT05029414)	Thrombectomy vs medical care	526	NIHSS ≥ 4 or disabling deficit; <6h from LKW or 6-24h from LKW if CT/CTP or DWI/FLAIR mismatch present; M2, M3-M4, A1, A2, A3, P1 or P2 occlusion	90 day-mRS	Switzerland	Dec/2024
ESCAPE-MeVO (NCT05151172)	Thrombectomy vs medical care	530	ASPECTS ≥ 8; NIHSS > 5 or NIHSS 3-5 with disabling deficit; <12h from LKW; M2-M3 or A2-A3 or P2-P3 occlusion; penumbra demonstrated by CTA/CT/clinical exam or CTP or MRP or DWI/MRA/clinical exam	90-day mRS	50 sites, coordination center at University of Calgary	Aug/2026
Vertobrobasilar occlusion						
BAOCHE (NCT02737189)	Thrombectomy vs medical care	318	pc-ASPECTS ≥ 6; NIHSS ≥ 6; 6-24h from LKW (isolated vertigo not considered); BA or intracranial VA occlusion	90-day mRS 0-3	China	December 2022
ATTENTION (NCT04751708)	Thrombectomy vs medical care	342	pc-ASPECTS ≥ 6 (≥ 8 if ≥ 80 years); NIHSS ≥ 10; <12h from estimated time of BAO	90-day mRS 0-3	China	May 2023
POST-ETERNAL (NCT05105633)	TNK 0.25 mg/kg vs tPA 0.9 mg/kg (+/- EVT)	688	pc-ASPECTS ≥ 7; <24h from LKW; BA occlusion (partial or complete)	90-day mRS 0-1 or return to baseline mRS	Australia	December 2026
Low NIHSS with proximal occlusion						
MOSTE (NCT03796468)	Thrombectomy vs medical care	824	ASPECTS ≥ 6; NIHSS ( 5; < 24h from LKW; ICA, M1 or M1-M2 occlusion	90-day mortality	France	Feb/2022
ENDOLOW (NCT04167527)	Immediate thrombectomy vs initial medical care	200	ASPECTS ≥ 6; NIHSS ( 5; < 8h from LKW; ICA, M1 or “M1-like” M2 occlusion	90-day mRS ordinal shift analysis and sICH	United States	Jan/2023

A1: indicates first segment of anterior cerebral artery; A2: second segment of anterior cerebral artery; A3: third segment of anterior cerebral artery; ASPECTS: Alberta stroke program early computed tomography score; BA: basilar artery; BAO: basilar artery occlusion; CT: non-contrast computed tomography; CTA: computed tomography angiography; CTP: computed tomography perfusion; DWI: diffusion-weighted magnetic resonance imaging; FLAIR: fluid-attenuated inversion recovery magnetic resonance imaging; ICA: internal carotid artery; IVT: intravenous thrombolysis; LKW: last known well; M1: first segment of middle cerebral artery; M2: second segment of middle cerebral artery; M3: third segment of middle cerebral artery; M4: fourth segment of middle cerebral artery; MRA: magnetic resonance angiography; MRP: perfusion magnetic resonance imaging; mRS: modified Rankin scale; NIHSS: National Institutes of Health Stroke Scale; P1: first segment of posterior cerebral artery; P2: second segment of posterior cerebral artery; P3: third segment of anterior cerebral artery; pc-ASPECTS: posterior circulation acute stroke prognosis early computed tomography score; sICH: symptomatic intracerebral hemorrhage; VA: vertebral artery.

## ENDOVASCULAR THROMBECTOMY FOR MEDIUM AND DISTAL VESSEL OCCLUSION

Occlusion of the middle cerebral artery (MCA) after its first bifurcation (although not anatomically accurate, the majority of thrombectomy trials considered this as M2 segment, so we will use this same terminology in this article) was excluded from most EVT randomized clinical trials, as well as anterior cerebral artery (ACA) and posterior cerebral artery (PCA) occlusions. Some of the trials included M2 occlusions, but they were under-represented and were mainly proximal and dominant trunks, functionally similar to M1 occlusions^18^.

Therefore, EVT for medium/distal vessels lacks support from studies with good evidence, despite the high prevalence of occlusion of these vessels in initial imaging (primary occlusions) accounting for 25-40% of strokes^19^ and also as a complication of large vessel occlusion treatment (secondary occlusion), either to the same territory of the initial large vessel (incomplete reperfusion was found in 80% of HERMES collaboration trials)^20^ or to a new territory (9.4% of internal carotid artery [ICA]/MCA thrombectomy complicates with ACA embolization)^21^.

As opposed to what is expected from proximal (ICA/M1) occlusion, distal vessel involvement confers a great heterogeneity of clinical presentations, as different territories are considered (ACA, MCA or PCA) and even when the same artery is occluded in two patients, due to great anatomic variability of distal vessels and collateral circulation, the functional and clinical importance of the occlusion can be diverse.

The occlusion diagnosis can be inferred as a dot sign in CT or MRI susceptibility vessel sign, confirmed when a cutoff can be shown in CT/MRI angiography. These signs can be more easily identified in the more proximal segments (M2, P1, A1), but can be difficult to find in more distal vessels and when there is a trifurcation or other anatomic variability, situations in which perfusion imaging can greatly improve sensibility to 80-100%^22^, as well as inform if the territory of the occluded artery is still viable. In the absence of perfusion imaging, a clinical deficit consistent with the occluded artery with a clinical/core (diffusion-weighted MRI) mismatch can also be indicative of viable tissue.

While smaller territories are at risk when compared to proximal occlusion (ICA/M1), a great proportion of patients remain functionally dependent (mRS >2): 60% of M2^23^, 70% of P1 and 44% of P2 occlusions^24^. ACA isolated occlusions were associated with 49% of patients moving to chronic care facilities or mRS >2^25^ and ACA embolization during ICA/M1 EVT conferred significant lower rates of 90-day mRS 0-2 (25% vs 48% [adjusted OR 0.48, 95% CI 0.25-0.92, P=0.027])^21^. Early recanalization after intravenous thrombolysis, despite greater than for ICA/M1 occlusion, are unsatisfactory, being achieved only in 37-44% of M2^26^ ^,^ ^27^, 52% of M2-M3^28^ and 42% of M3/ACA/PCA occlusions^27^, justifying the study of alternative treatments, as EVT. 

Due to greater distance, increased tortuosity and smaller diameters in more distal vessels, EVT poses more technical challenges and risks (dissection, perforation, vasospasm, embolization to new territories) when compared to proximal occlusions, but endovascular technology has advanced with smaller/low profile stent retrievers and aspiration catheters that are suitable for distal vessels^29^ ^-^ ^30^.

The HERMES collaboration analyzed the data from 130 patients with M2 occlusion included in the pivotal EVT trials and found a TICI 2b-3 recanalization in 59.2% of patients and 90-day mRS 0-2 in 58.2% of EVT group against 39.7% in control group (adjusted OR 2.39 [95% CI 1.08-5.28, p=0.03]). Treatment effect favoring EVT was maximal in proximal and dominant M2 (adjusted OR of 2.68 and 4.08, respectively)^18^. Thrombectomy with stent retrievers and aspiration catheters has shown comparable results for M2 occlusions in more recent studies, with a TICI 2b-3 recanalization rate above 80%^31^.

Small (n=69 and 130) single center observational studies of EVT for M3-M4, ACA and PCA occlusions (distal MCA cases were majority) showed that the procedure is feasible and safe, with satisfactory recanalization in 75-83% of cases, with similar results between primary and secondary occlusions and relevant intraparenchymal bleeding in 7-8% of cases^32^ ^,^ ^33^. In one of the studies, which used several techniques (stent-retriever in 54%, aspiration in 45% and intra-arterial recombinant tissue plasminogen activator [rtPA] in 52%), the rate of 90-day mRS 0-2 was 30% (38-54% for M3), but strokes were moderate/severe, with median baseline NIHSS of 18 (IQR 13-23) and mortality of 20%^32^.

A multicenter case-control study of EVT for primary distal PCA (P2-P3) occlusion (TOPMOST) showed a mTICI 2b-3 recanalization rate in 87.4% of cases using several techniques (stent-retriever +/- aspiration in 72%, primary aspiration in 26% and intra-arterial rtPA in 1.4%). Among 184 matched patients, EVT conferred a non-significant better result in median NIHSS decrease at discharge (mean difference -1.5 [95% CI 3.2 to -0.8; P=0.06]), with significant results shown in subgroup analysis of NIHSS ≥ 10 (mean difference −5.6 [ 95% CI −10.9 to −0.2; P=0.04) and patients not submitted to intravenous rtPA (mean difference −3.0 [95% CI −5.0 to −0.9; P=0.005). Although the favorable results of NIHSS decrease at discharge, no difference was shown for 90-day mRS 0-2 (76.6% vs 75.4%, P=0.87). Regarding safety concerns, there was no difference in sICH (4.3% in both groups, P>0.99) or mortality (11.8% vs 15.8%, P=0.40). Median baseline NIHSS in this study was 5 (IQR 3-10)^34^. 

Small (n=41 and 30) single center observational studies of EVT (83% with stent-retriever) for distal ACA occlusions also showed good mTICI 2b-3 rates (83-88%) and low rates of complications, with 10% of asymptomatic regional subarachnoidal bleed described in one series and 10% of vasospasm and 3.3% of asymptomatic hemorrhagic transformation in ACA territory in the other. Initial NIHSS was high (mean of 17-18) and 90-day mRS 0-2 was achieved in only 20-36% of patients, but almost all were associated with ICA/M1 occlusion, limiting the interpretation of these outcomes^35^ ^,^ ^36^.

Although several studies indicate that thrombectomy for distal occlusion is safe and viable, only a few studies have compared its efficacy to intravenous rtPA. One meta-analysis of four retrospective observational studies (with significant heterogeneity [I^2^=89%]) with 381 patients with A2, M3-M4 or P2-P4 occlusion did not demonstrate a significant difference in 90-day mRS 0-2 between the groups (OR 1.16, 95% CI 0.23-5.93; P=0.861)^37^.

Currently there is very limited evidence, mainly from observational studies, regarding endovascular thrombectomy to distal and medium vessels occlusion, making it not possible to make general recommendations about its clinical effectiveness in comparison with best medical care. In selected cases, considering the relative safety and high recanalization rates of present-day endovascular techniques, a multi-specialty (stroke neurologist, neuroradiologist and neurointerventional radiologist) individualized approach could be sought, taking several factors into consideration: more proximal occlusions (A1, P1 and M2 - especially if the occluded M2 is dominant, for which there is some subgroup favorable data from randomized clinical trials), higher baseline NIHSS or disabling deficits, presence of relevant penumbra-core or clinical-core mismatch in eloquent locations, contraindication to intravenous thrombolysis (IVT) as factors in favor of EVT; and difficult proximal access, more tortuous vasculature, lower baseline NIHSS with no disabling deficits, greater proportion of core in the involved territory as factors against it. A lot of questions still need to be addressed, as the use of advanced imaging techniques in selection of patients; the role of intra-arterial rtPA, which was demonstrated to be safe^38^ and with some exciting good clinical results in a recent randomized trial suggesting the importance of distal circulation reperfusion^39^; what the factors are for achieving a relevant treatment effect; among others. Despite the difficulty of designing randomized clinical trials due to great clinical heterogeneity, several are under way and some answers are expected in the future (Table 1).

## ACUTE BASILAR ARTERY OCCLUSION

Acute ischemic stroke due to basilar artery occlusion (BAO) represents 20% of posterior circulation strokes and 1-4% of all ischemic strokes^40^ ^,^ ^41^. BAO is one of the most challenging emergencies with death rates up to 80-90% in the absence of therapeutic interventions. The clinical picture of BAO varies greatly, with 30-60% of patients presenting with coma, tetraplegia or locked-in syndrome^41^. In the majority trials of stroke reperfusion, BAO and posterior circulation stroke are underrepresented^40^. Optimal reperfusion strategies are still under discussion.

The Basilar Artery International Cooperation Study (BASICS) registry, an observational prospective multicenter study, reported a 10-19% absolute lower death rate and dependency for severe patients with acute BAO treated with intravenous thrombolysis (IVT) or EVT, compared to therapy with antiplatelet or anticoagulant drugs. Yet death ranged from 40% to 50% in patients submitted to IVT and EVT, with 6% of sICH in IVT group^42^.

The reverse filling of the distal basilar artery by posterior communicating arteries and the abundant collaterals from posterior circulation collaborate to brainstem ischemic resistance. It has been suggested that as long as perforators are patent, brainstem can be viable for a long period. The small infarct cores compared with anterior circulation contribute to resistance to hemorrhagic transformation in the brainstem^43^. Based on these hypotheses, IVT has been simultaneously or immediately followed by anticoagulation in BAO for more than 20 years by a group from Helsinki in patients up to 12 hours after the sudden onset of neurological deficits or up to 48 hours of progressive neurologic deficits^44^. The Helsinki group reported that, in 207 patients treated with IVT and anticoagulation, favorable outcomes of mRS 0-3 were achieved in 41.1% and mRS 0-2 in 31.3% at three months. The recanalization rate was around 70% and sICH, 11.4%^45^. However, there is no evidence based on randomized clinical trials that add-on anticoagulation is beneficial in acute BAO.

EVT has led to dramatic changes in anterior circulation stroke treatment and a meta-analysis of 45 studies and 2056 patients concluded that recanalization has a significant effect on BAO outcomes (number needed to treat of 2.5 to prevent death and disability)^46^. Rates of recanalization up to 80-100% can be achieved after EVT in BAO. Yet, no striking benefit has been achieved in the latest randomized trials of EVT in BAO. Reasons that may justify this are the slow recruitment, lack of a criteria for patient selection such as in anterior circulation, lack of equipoise, as there are several reports of a favorable outcome with EVT in retrospective and observational trials, so physicians may consider it is not appropriate to randomize these patients anymore. 

An open label, randomized trial (BEST) of EVT up to eight hours after the estimated onset of BAO was stopped prematurely due to a high crossover rate (22%) and drop in recruitment, probably due to loss of equipoise. The study’s primary endpoint analysis failed to show a difference in the proportion of patients with favorable neurologic outcomes (90-day mRS 0-3 of 42% in the intervention group vs 32% in controls [adjusted OR 1.74; 95% CI 0.81-3.74]) despite 71% achieving successful reperfusion. After accounting for the effects of crossover, there were higher rates of favorable outcome in patients who received intervention compared to control in the as-treated population, 47% vs 24% (adjusted OR 3.02; 95% CI 1.31-7.00). A higher incidence of sICH in the intervention group was documented (8% vs 0), however mortality was similar (33% in intervention vs 38% in control)^47^.

BASILAR, a nonrandomized study, suggested that EVT (n=647) may be safe and effective up to 24 hours after the estimated onset of BAO, when compared to control (n=182). EVT was associated with a significantly higher rate of 90-day mRS 0-3 (adjusted OR 4.70; 95% CI 2.53-8.75) and a lower mortality rate at 90 days (adjusted OR 2.93; 95% CI 1.95-4.40) despite an increase in sICH (7.1% vs 0.5%)^48^.

BASICS, a randomized trial of EVT up to six hours after the estimated onset of BAO, failed to demonstrate an overall benefit of EVT compared with control. The primary endpoint of favorable functional outcome (mRS 0-3) at 90 days was not significantly different between the groups of EVT (44.2%) and control (37.7%). In subgroup analysis, patients with NIHSS ≥10 had significantly more benefit if treated with EVT. EVT group sICH rate was 4.5%. The slight benefit of EVT in BASICS trial might be related to the overwhelming effect of IVT, administered to 80% of the patients and selection bias of patients to be randomized^49^. Comparisons between BASICS, BASILAR and BEST trials are limited by patient heterogeneity, differences in medical treatment (20-30% of IVT in BEST and BASILAR) and different time spans allowed for reperfusion. A Bayesian meta-analysis of both randomized trials and a patient-level meta-analysis of NIHSS ≥ 10 from both trials suggested significantly improved outcomes in the EVT arm^50^ ^,^ ^51^.

Relevant variables may influence BAO outcome. These are: onset time to treatment, age >60 years, higher NIHSS and Glasgow coma scale scores at admission, failure to recanalize and collateral circulation^52^ ^-^ ^54^. Embolic etiology may also be associated with worse outcomes. Nevertheless, higher recanalization rates have been reported with embolic occlusion than with in situ atherosclerotic thrombosis in BAO^55^. The site of occlusion has also has prognostic implications when treated, and distal lesions have better outcomes than proximal ones^56^.

The extent of ischemia can be assessed with the posterior circulation acute stroke prognosis early CT score (pc-ASPECTS)^57^. Patients with pc-ASPECTS <8 typically have worse functional outcomes than those with higher scores, despite recanalization. However, others found that lower thresholds of pc-ASPECTS (≥5) are also associated with a good outcome^58^ ^-^ ^60^. In patients with pc-ASPECTS ≥8, the time to treatment may not influence outcomes^44^. In addition, in the ETIS study^59^, a time-dependent benefit was found in patients with pc-ASPECTS <8. In BASICS registry^54^, most of the benefit of reperfusion therapies was time-dependent, no severe patient after nine hours of estimated BAO achieved a favorable outcome. The pc-ASPECTS has been criticized by its interrater variability, need for expertise in scoring, and limitations of assessment of the posterior fossa on CT^57^. 

Besides pc-ASPECTS, cerebellar infarct volume also seems to be an independent predictor for 90-day mortality. It is known that cerebellar mass effect due to infarction and edema may lead to hydrocephalus and brainstem compression. It is one of the important causes of death in acute BAO patients. In one study, risk of death was increased for baseline volume ≥4.7 ml^61^.

Collaterals and thrombus burden are an important predictor of clinical outcomes in anterior and posterior circulation strokes. BATMAN is a 10-point scoring system that includes these characteristics and is considered a predictor of functional outcome in BAO^62^. Higher BATMAN score means shorter thrombus and/or better collaterals. Reperfusion was associated with a good outcome in those with favorable BATMAN score (≥7), despite time to treatment <6 or >6 hours. Yet, in patients with unfavorable BATMAN, time to treatment <6 hours was significantly related to good outcomes^52^.

There are many reports of patients with BAO, low pc-ASPECTS, long onset to treatment time (>24 hours) and severe presentation with favorable functional outcomes after reperfusion treatment and intensive rehabilitation. This is an additional dilemma for decision-making because despite a predicted unfavorable prognosis, some patients can achieve functional independence^63^ ^,^ ^64^. Therefore, physicians must be cautious with the self-fulfilling prophecy in BAO therapy decisions.

Therefore, against a life-threatening condition such as BAO, the best choice is an individualized therapy for each patient. Neurologists should decide on the type of treatment combination (IVT, EVT, add-on anticoagulation or no reperfusion therapy) based on clinical features (age, NIHSS, Glasgow Coma Scale), radiological appearance (infarct core, pc-ASPECTS, BATMAN, cerebellar infarct volume, site of occlusion), onset time to treatment, stroke mechanism and risk of sICH. The better the combination of these characteristics probably the better the outcome will probably be. If the combination of the variables does not seem favorable, most likely the recanalization will be futile. 

The next studies in BAO should focus on how to adequately select patients who can have a substantial treatment effect and achieve good outcomes.

## PROXIMAL OCCLUSION WITH LOW NIHSS SCORES

Patients with mild strokes can worsen after initial evaluation, mainly secondary to ischemic complications probably due to failure of collateral circulation and the presence of proximal occlusion is an independent predictor for this adverse scenario^65^. Around 10% of patients with baseline NIHSS <6 have proximal occlusion detected on CT/MRI angiography and 20% have clinical deterioration ≥ 4 point on NIHSS, usually early after hospital arrival (median of 3.6h [IQR 1-16h]), causing worse functional outcomes^66^. Therefore, these patients should be transferred for an EVT-capable center and closely monitored. 

Thrombectomy trials included in HERMES collaboration included a small number of patients with NIHSS ? 10 and subgroup analysis for this population was not powered to show benefits from EVT^1^. Until now, there are no randomized trials on this topic and observational evidence is divergent.

Some multicenter observational studies found no difference in functional outcomes between EVT group and best medical care group, with a higher risk of sICH in EVT group. However, in these studies, there was no differentiation if patients received immediate EVT on admission or rescue EVT (performed only after clinical deterioration); both were included in the EVT group and the prognosis of patients that have clinical deterioration is known to be worse. In the studies in which IVT to EVT time was available, median time was much longer than would be expected^67^ ^,^ ^68^. In a planned randomized trial, patients that received initial medical care followed by rescue EVT would be considered as being part of the medical care group.

A multicenter retrospective study considered this important factor in its analysis, including 80 patients in the immediate EVT group and 220 patients in the medical care group, of which 25 (11.3%) received rescue EVT. Immediate EVT was an independent predictor of mRS 0-2 (OR 3.1, 95% CI 1.4-6.9) with an absolute difference of 15% (85% vs 70%), but conferred not statistically significant higher rates of sICH (5% vs 1.4%, P=0.08). Matched analysis corroborated the results, with higher rates of 90-day mRS 0-2 in the immediate EVT group (84.4% vs 70.1%, P=0.03) and not statistically significant higher numbers of sICH (5.2% vs 2.6%, P=0.41)^69^.

Despite current guidelines recommending against IVT in minor non-disabling strokes, this recommendation was mainly based on the results of the PRISMS trial, in which the rate of proximal occlusion was a minority^70^. Therefore, due to the increased risk of stroke progression in the patients with proximal occlusion, IVT should still be considered.

A recent multicenter retrospective French study with 729 patients with large vessel occlusion and NIHSS <6 that were treated with IVT showed that still after IVT ischemic early neurological deterioration occurred in 12% of patients and was strongly associated with poorer 90-day outcomes, even in patients who underwent rescue thrombectomy. This cohort was used to elaborate a novel 4-point score for early ischemic neurological deterioration prediction using the two factors that were independently associated with it in multivariable analysis: a more proximal occlusion site (0 point for M2; 1 point for distal M1; 2 points for proximal M1 or tandem or basilar; 3 points for terminal ICA) and longer thrombus (1 point if ≥ 9mm). The score was validated in another cohort and showed good discriminative power. In both cohorts early deterioration probability was approximately 3%, 7%, 20%, and 35% for scores of 0, 1, 2 and 3-4, respectively^71^.

While the results of two ongoing randomized clinical trials are expected in the next year to better guide reperfusion therapies (Table 1), in conjunction with the imaging features of the aforementioned score, some clinical information that possibly indicates an unstable collateral circulation with higher risk of imminent failure can be used to help in making clinical decisions, such as deficit fluctuation, auto-hypertension and a stress test consisting of sitting the patient upright for 10-20 minutes or even walking and observing for signs of clinical deterioration. Some clinicians may also pursue surrogate measures of vascular reserve such as transcranial doppler ultrasound or perform perfusion imaging to demonstrate a possible territory at risk^72^. Although biologically plausible, all these alternatives lack proper validation.

## INTRAVENOUS THROMBOLYSIS IN PATIENTS USING DOACS

In the last few years, there has been an increase in the number of patients taking direct oral anticoagulants (DOACs). The American Heart Association guidelines recommend against IVT in patients who have taken DOACs in the last 48 hours. However, there have been successful case reports of patients receiving IVT after reversal of dabigatran by idarucizumab^73^. Other reversal agents such as andexanet alpha have potential prothrombotic effects, but its reversal effects are known to be less reliable, and the evidence is even scarcer. A meta-analysis (with no heterogeneity, I^2^=0) of patients taking DOACs (n=366) that received IVT found no difference in sICH compared to patients taking warfarin with international normalized ratio < 1.7 (n=2133) or patients without prior anticoagulation (n=50324). In addition, last DOAC intake time and use of pre-thrombolysis idarucizumab were not related with sICH^74^. However, randomized trials are still lacking and are not expected soon. Unaltered drug-specific coagulation assays or thromboelastography could serve as a surrogate for low DOAC activity and confer a better safety profile for thrombolysis, but these are not readily accessible in a timely manner in most hospitals, validation for this use has yet to be reported and patients with altered results would still be excluded from treatment. 

Factors that could counterbalance in favor of administering IVT would be a severe/debilitating presentation, possibility of IVT in the first hours when benefit is maximal, absence of other conditions that increase sICH risk, longer time from last DOAC administration and normal drug-specific assays/thromboelastography. In patients with minor stroke, EVT indication (where IVT bridging efficacy is under debate), high risk for sICH, >3h from last known well and very recent use of DOAC, the risks would probably outweigh the benefits of IVT. Further studies are urgently needed to access whether previous DOAC use should be withheld as a contraindication in current guidelines, as has occurred with several previous IVT contraindications. The development of more accessible point of care coagulation tests for better selection of patients might also be a useful strategy in the near future^75^.

## ENDOVASCULAR THROMBECTOMY AFTER 24 HOURS OF TIME LAST KNOWN WELL

EVT up to 24 hours had a huge effect and shifted the traditional time window to tissue-based evaluation for patient selection. A retrospective multicenter series demonstrated similar outcomes in patients treated with more than 24 hours from time last known well that otherwise met DAWN^76^ criteria in comparison with patients in DAWN trial intervention group regarding mTICI 2b-3 (81% vs 84%, P=0.72), 90-day mRS 0-2 (43% vs 48%, P=0.68) and sICH (5% vs 6%, P=0.87). Median interval from time last known well to groin puncture was of 48 hours (IQR 30-72h)^77^. Another retrospective series of patients with more than 16 hours from time last known well (median of 43.5h [IQR 23-77h]) where EVT was indicated at the discretion of the clinician (n=24) showed favorable outcomes when compared to a propensity score-matched data set with a higher proportion of 90-day mRS 0-2 (adjusted OR 11.08 [95% CI 1.88-108.60]. Although not statistically significant, DEFUSE-3^78^ imaging criteria might have the potential to determine which patients benefit from treatment^79^.

There are probably few patients who are ultra-slow progressors due to enhanced collateral circulation that still can benefit from EVT. Therefore, reperfusion strategies might be considered as a possibility even in patients beyond 24 hours of time last known well with small infarct core at presentation and who would otherwise be included in DAWN or DEFUSE-3 criteria.

In conclusion, the strong evidence in favor of reperfusion therapies in the management of acute ischemic stroke and its relevant impact on functional outcomes for some already established situations have led to research as to whether other, not yet still well-studied stroke populations could also greatly benefit from it. Fortunately, stroke research keeps progressing with outstanding multicenter and coordinated efforts to advance the boundaries of current knowledge, with exciting new data expected in the near future (Table 1).
